# Data driven surrogate signal extraction for dynamic PET using selective PCA: time windows versus the combination of components

**DOI:** 10.1088/1361-6560/ad5ef1

**Published:** 2024-08-14

**Authors:** Alexander C Whitehead, Kuan-Hao Su, Elise C Emond, Ander Biguri, Ludovica Brusaferri, Maria Machado, Joanna C Porter, Helen Garthwaite, Scott D Wollenweber, Jamie R McClelland, Kris Thielemans

**Affiliations:** 1 Institute of Nuclear Medicine, University College London, London, Greater London, United Kingdom; 2 Centre for Medical Image Computing, University College London, London, Greater London, United Kingdom; 3 Department of Computer Science, University College London, London, Greater London, United Kingdom; 4 Molecular Imaging and Computed Tomography Engineering, GE Healthcare, Waukesha, WI, United States of America; 5 Department of Applied Mathematics and Theoretical Physics, University of Cambridge, Cambridge, Cambridgeshire, United Kingdom; 6 Computer Science and Informatics, London South Bank University, London, Greater London, United Kingdom; 7 Centre for Respiratory Medicine, University College London, London, Greater London, United Kingdom

**Keywords:** PET, dynamic PET, surrogate signal extraction, PCA, data driven gating

## Abstract

*Objective.* Respiratory motion correction is beneficial in positron emission tomography (PET), as it can reduce artefacts caused by motion and improve quantitative accuracy. Methods of motion correction are commonly based on a respiratory trace obtained through an external device (like the real time position management system) or a data driven method, such as those based on dimensionality reduction techniques (for instance principal component analysis (PCA)). PCA itself being a linear transformation to the axis of greatest variation. Data driven methods have the advantage of being non-invasive, and can be performed post-acquisition. However, their main downside being that they are adversely affected by the tracer kinetics of the dynamic PET acquisition. Therefore, they are mostly limited to static PET acquisitions. This work seeks to extend on existing PCA-based data-driven motion correction methods, to allow for their applicability to dynamic PET imaging. *Approach.* The methods explored in this work include; a moving window approach (similar to the Kinetic Respiratory Gating method from Schleyer *et al* (2014)), extrapolation of the principal component from later time points to earlier time points, and a method to score, select, and combine multiple respiratory components. The resulting respiratory traces were evaluated on 22 data sets from a dynamic [^18^F]-FDG study on patients with idiopathic pulmonary fibrosis. This was achieved by calculating their correlation with a surrogate signal acquired using a real time position management system. *Main results.* The results indicate that all methods produce better surrogate signals than when applying conventional PCA to dynamic data (for instance, a higher correlation with a gold standard respiratory trace). Extrapolating a late time point principal component produced more promising results than using a moving window. Scoring, selecting, and combining components held benefits over all other methods. *Significance.* This work allows for the extraction of a surrogate signal from dynamic PET data earlier in the acquisition and with a greater accuracy than previous work. This potentially allows for numerous other methods (for instance, respiratory motion correction) to be applied to this data (when they otherwise could not be previously used).

## 1. Introduction

Respiratory motion reduces image resolution, by introducing blurring, as well as misalignment artefacts in positron emission tomography (PET) (Nehmeh and Erdi 2008). Methods of motion correction, such as gating, are commonly based on a surrogate signal (SS), see Kyme and Fulton (2021), Lamare *et al* (2022) for recent reviews. This SS is a respiratory trace which reflects the position of the anatomy of the patient in the respiratory cycle over time (Kesner and Kuntner 2010, Kesner *et al* 2013).

Initial research concentrated on methods that determine the SS via an external device. For instance, a spirometer (Voscopoulos *et al* 2013), a belt (Yu *et al* 2016), an imaging device such as a depth sensing camera (Xia and Siochi 2012, Silverstein and Snyder 2018), or the real time position management (RPM) (Bettinardi *et al* 2013). However, methods utilising external devices suffer from several disadvantages, such as drift and/or requiring a constant line of sight. Furthermore, most external device methods can only track the surface deformations of the patient, as opposed to the internal ones. Finally, such methods require the use of additional equipment, a change to clinical practise, and must be acquired alongside any other data, not retrospectively.

Thus, PET data driven (DD) methods to extract the SS, which do not require additional equipment and can be applied retrospectively, have become an alternative for static PET data (Kesner *et al* 2014). DD methods include, but are not limited to, those which attempt to spatially track aspects of the acquisition data, be that reconstructed or not, or ones which use methods such as dimensionality reduction (Lamare *et al* 2022). We briefly discuss these methods here, but also see section 2.

Some DD solutions make use of aspects of the image acquisition itself, by reconstructing short time frame images and tracking regions of them over time. Such as, an external or inserted radioactive fiducial marker (Zimmermann *et al* 2003, Büther *et al* 2013), a tumour (Bundschuh *et al* 2007), or a combination of patterns from different voxels (Kesner *et al* 2009). Some methods make use of magnetic resonance (MR) information (tracking the position of the diaphragm using a pencil shaped navigator) (Taylor *et al* 1997, Fürst *et al* 2015). A disadvantage of image space based methods, is computation time and potential poor quality due to low count statistics. Obviously, methods which require the insertion of objects into a patient have the unnecessary side effect of causing harm to the patient. Furthermore, to make use of MR information requires a combined PET/MR.

Alternatively, aspects of the data in sinogram space can be individually tracked directly from the list mode data, such as the centroid of distribution (COD) or centre of mass (COM) (Klein *et al* 2001, Bruyant *et al* 2002, Ren *et al* 2017, Feng *et al* 2018). A potential disadvantage, is that these methods require structures with high contrast in sinogram space. A final class of methods, uses short time frame sinograms (often at low spatial resolution) and detects motion patterns in the whole sinogram. Such methods rely on the fact that, in static PET, the main cause of (non-stochastic) change in the data is motion. The main sinogram-based methods are the spectral analysis method (SAM) method (Schleyer *et al* 2009, 2011, 2014), the sinogram region fluctuation (SRF) method (Kesner and Kuntner 2010), and a method based on principal component analysis (PCA) (Thielemans *et al* 2011, Bertolli 2018), briefly described below.

• SAM identifies regions in sinograms which are likely to be experiencing respiratory motion. This is achieved by analysing the frequency spectrum of the result of applying a fast Fourier transform (FFT) to each bin in the sinogram. A bin which is experiencing respiratory motion will have a peak in the frequency spectrum at the frequency of the respiratory motion. Through this, areas in the sinogram which are experiencing respiratory motion are determined, and the total number of counts in these regions, over time, is used to estimate a SS (Schleyer *et al* 2009, 2011, 2014).
• SRF proposes to recursively combine signals from sinogram bin time activity curves (TACs). This is performed using a score based on the ratio between respiratory and non-respiratory content (with a positive and negative sign) in order to maximise its standard deviation (Kesner and Kuntner 2010). However, a disadvantage of the use of standard deviation, as the objective, would be that there are many ways to increase standard deviation, which are not acquiring a better respiratory trace. For instance, noise may increase the standard deviation of a signal.
• PCA works similarly to singular value decomposition (SVD), in fact most implementations of PCA use SVD. The goal of this method is to find linear transforms of the data, such that it is projected to a space along which its axis point in the direction of greatest variance (and then second greatest variance etc). For SS extraction PCA is applied across a time series of sinograms. The weighting of each principal component (PC) for each time point would be the signal. Generally multiple PCs are extracted, and the one which contains the most respiratory information (determined using FFT) is selected (Thielemans *et al* 2011, Bertolli 2018).

To-date, evaluations of these methods have been almost exclusively performed on static PET data, mostly using Fluorine-18 Fludeoxyglucose ([^18^F]-FDG). They include comparisons with external devices (such as the RPM), MR navigator based SSs (Manber *et al* 2015), as well as image quality (Walker *et al* 2019, Büther *et al* 2020). Preliminary investigations indicated that many sinogram-based methods all perform similarly (Thielemans *et al* 2013).

However, current DD methods are adversely affected by the radiotracer kinetics of a dynamic acquisition, where the tracer is injected after the beginning of the scan. As an example, methods that use dimensionality reduction (such as PCA) are hampered by the fact that at the start of the scan, rapid redistribution of the radiotracer (rather than the respiratory motion) causes more variance in the data. Previously, work was performed to extend the SAM method to be robust to radiotracer kinetics. This work proposed the use of short time Fourier transform (STFT) to generate masks for SAM (rather than a static mask for all time intervals), this was called kinetic respiratory gating (KRG) (Schleyer *et al* 2014). STFT operates by splitting the data into windows, and applying a FFT on them independently. This could be approximated, by windowing the data first, and then performing SAM. However, this method was unable to extract a usable signal at very early time intervals (after tracer injection).

The aim of the current work is to propose several adaptions of the PCA method, with which it can be used with dynamic data, and compare their performance with a method based on KRG. The methods explored in this work include; the use of a moving window, re-use of the PCs from a later time interval to estimate the SS from earlier time intervals, and the automatic scoring, selection, and combination of multiple PCs, akin to SRF.

Firstly, in section 2, the data (including train and test splits) will be introduced, before moving on to the methods which are being proposed or compared. Next, in section 3, a more thorough description of the data, including how it is prepared, and the evaluation methods used to compare the methods are presented. In this section we also introduce potential post-processing techniques, which can be performed to SSs, to improve results generally. Followed by, in section 4, figures depicting a comparison of the methods defined in the previous section are shown and discussed. The advantages and disadvantages of the methodology of the work presented, is highlighted in section 5. Finally, in section 6, the arguments put forth are drawn together, before briefly pointing out the potential future directions for the work.

## 2. Methods

Here, we briefly describe the data acquisition, before describing methods that are either simple modifications of the conventional methods (based on KRG) or use a novel method to score, select, and combine signals. These methods can be used with SAM, but for simplicity, we will refer specifically to PCA.

### 2.1. Data acquisition and train/test split

Data used was acquired from a research study with patients suffering from idiopathic pulmonary fibrosis (IPF) (Emond *et al* 2020). 22 dynamic [^18^F]-FDG acquisitions, with a field of view (FOV) covering the upper lung and heart, were acquired on a General Electric (GE) Discovery 710 in list-mode. Data used in this study was from the first 14 min with the acquisition starting roughly 20 s before injection of the radiotracer. An external SS was acquired in parallel using an RPM (Oh *et al* 2019).

**Figure 1.**
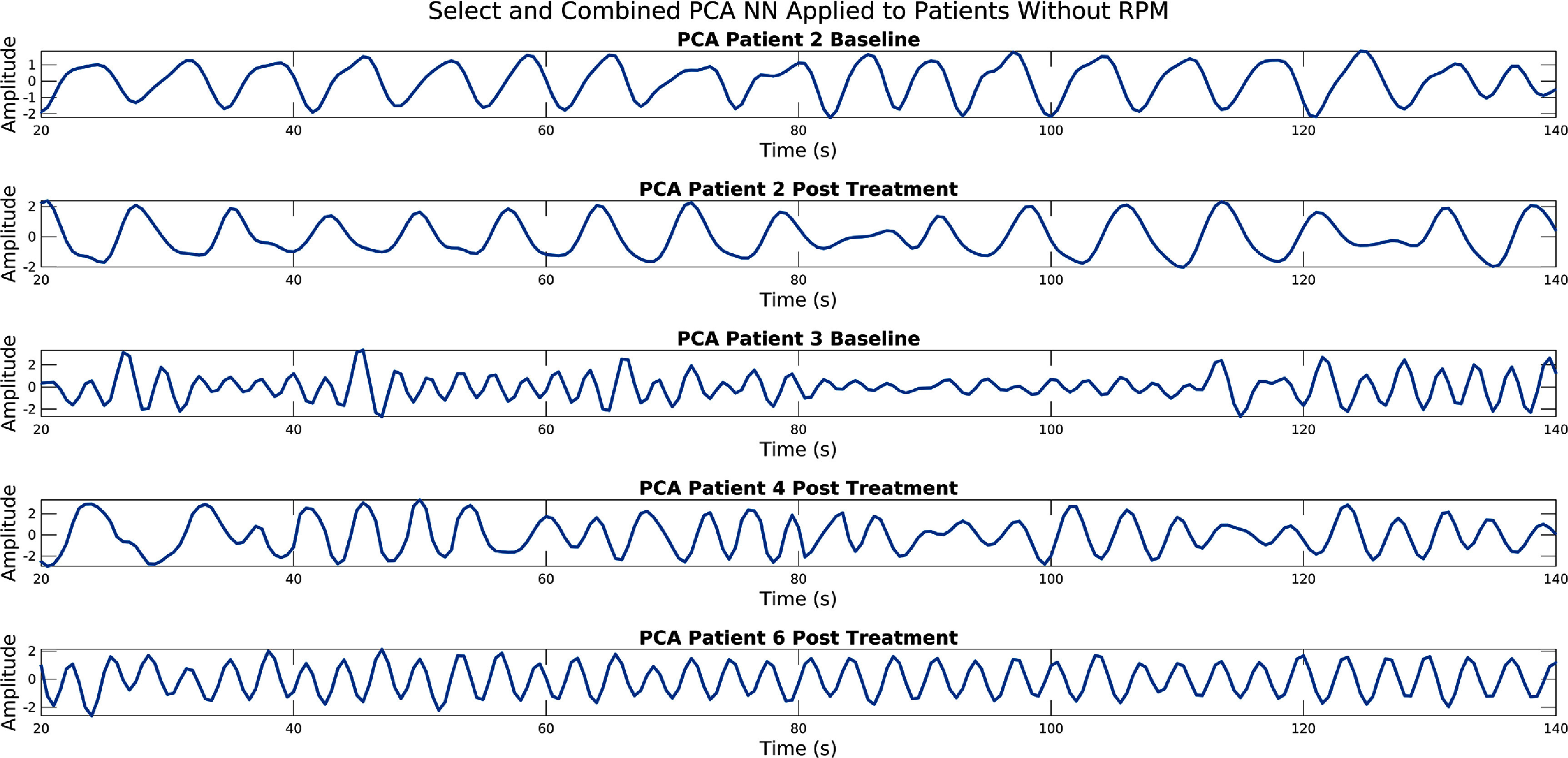
Signals for the first usable 120 s (between 20 s and 140 s) generated using the Score, Select, and Combine NN based scoring method. This is for the five acquisitions which did not have a usable RPM signal and as such could not be used for training or testing.

These 22 acquisitions comprised of two scans each of 11 subjects. One scan was performed before and one scan was performed some time after an intervention was performed. In this case, the intervention was the administration of an anticoagulant drug. The first acquisition of each patient is called the baseline scan and the second acquisition is called the post treatment scan (Emond *et al* 2020). Of these 22 acquisitions only 10 were suitable to be used as either part of the training or testing process. Five of the acquisitions could not be used as the list-mode itself could not be loaded by the software. The remaining acquisitions could not be used for either training or testing due to issues with the acquisition of the RPM. For instance, the acquired RPM could not be synced with the list-mode. These data without RPM could be used with the subsequent methods, just not evaluated, as can be seen in figure 1.

To form a train and test split the data was randomly split into three training data points and seven testing data points. More than one training data point was selected to attempt to prevent overfitting on a single data point. Data points from both baseline and post treatment acquisitions were added to prevent overfitting on type of acquisition. Train data points were selected such that the same patient did not appear in both the train and test dataset, to attempt to mitigate data leakage. A validation dataset was not utilised due to the low number of data points.

For selection of hyperparameters, the method was applied to the train dataset, and the correlation coefficients with RPM were computed. The mean of the three resulting correlation coefficients was maximised while varying a hyperparameter.

### 2.2. Conventional PCA

As described above in section 1, PCA is applied to the entire data set in one go, as it would be if the data were from a static acquisition. The PC containing the respiratory trace may not be the first one. Generically, a number of PCs are selected, and the signal that maximises a score with the appropriate respiratory features (for instance, a frequency matching the common human breathing), is selected as the SS (Bertolli *et al* 2017). In this implementation, when selecting from a number of PCs the one which contains respiration, the signal which maximises the score seen in the algorithm 1 is used. For reference, power spectral density (PSD) being the result of decomposing the signal into discrete frequency bins, using FFT, where the power is the energy at that frequency.

**Table:** 

**Algorithm 1.** Conventional score.
**Data:** *timeSeriesSinograms*, *PC*, *respiratoryFrequencyWindow*
**Result:** *respiratoryScore*
**1** **for** *sinogram* in *timeSeriesSinograms* **do**
**2** *respiratorySignal* append *sinogram* × *PC*
**3** **end**
**4**
**5** *PSD* = absolute of FFT on *respiratorySignal*
**6**
**7** *respiratoryScore* = mean value of *PSD* within *respiratoryFrequencyWindow*

The generic equation for calculating the weights (or signal) from the PC and data is

\begin{equation*} W = PC \times S\end{equation*}

where in equation (1) *PC* represents the PC (which in this case is the shape of one sinogram) and *S* represents a time series of sinograms. × denotes element-wise multiplication of the arrays (in this case multiplication of the one PC by each sinogram in the time series *S*), followed by summing. In fact, a similar equation is used by SAM, where a ‘signed mask’ is multiplied with the data and summed.

### 2.3. Moving window method

As shown in the algorithm 2, the data is split into a series of windows, where each subsequent window overlaps with the previous window by half its length. The motivation for attempting the Moving Window method, is to increase the relative importance of motion vs kinetics. This is achieved through small windows being used at early time intervals, where the radiotracer kinetics are at their most severe, and longer windows can be used at later time intervals to reduce noise. If SAM is used rather than PCA, then the method approximates KRG (Schleyer *et al* 2014).

**Table:** 

**Algorithm 2.** Moving window method.
**Data:** *timeSeriesSinograms*, *windowSizes*
**Result:** *respiratorySignal*
**1** *index* = 0
**2** *whileBool* = true
**3**
**4** **while** *whileBool* **do**
**5** **if** *index* > length of *timeSeriesSinograms* **then**
**6** *index* = length of *timeSeriesSinograms* - *windowSize*
**7** *whileBool* = false
**8**
**9** set *windowSize* to value at *index* of *windowSizes*
**10**
**11** *windowSignal* = fill with Not a Numbers (NaNs) to *index*
**12** *windowSignal* append compute PC weight with PCA for data between *index* and *index* + *windowSize*
**13** *windowSignal* append NaNs to length of *timeSeriesSinograms*
**14**
**15** **if** *windowSignal* correlation with last *signal* in *signals* $\lt$ 0.0 **then**
**16** *windowSignal* = *windowSignal* $\times -1.0$
**17**
**18** *signals* append *windowSignal*
**19**
**20** *index* = *index* + $\dfrac{\textit{windowSize}}{2}$
**21**
**22** **end**
**23**
**24** *respiratorySignal* = mean of *signals* ignoring NaNs

**Figure 2.**
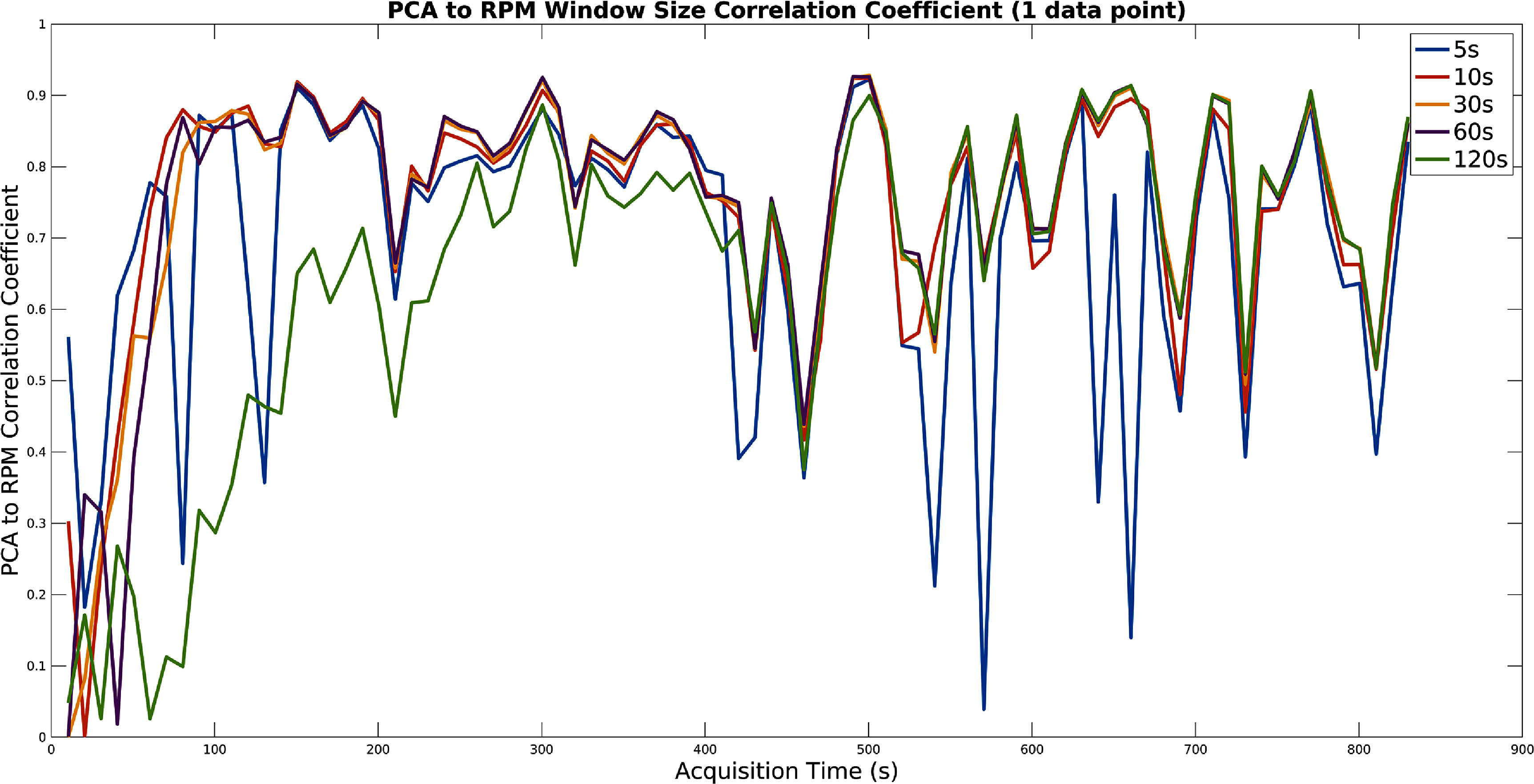
A plot showing an example of the influence of the moving windows size for the PCA method. For different fixed window sizes, the correlation of the extracted signal to the RPM is shown for the windows sliding over the whole acquisition (shown for the first acquisition of patient one). Note that 0.5 s time frames were used.

**Figure 3.**
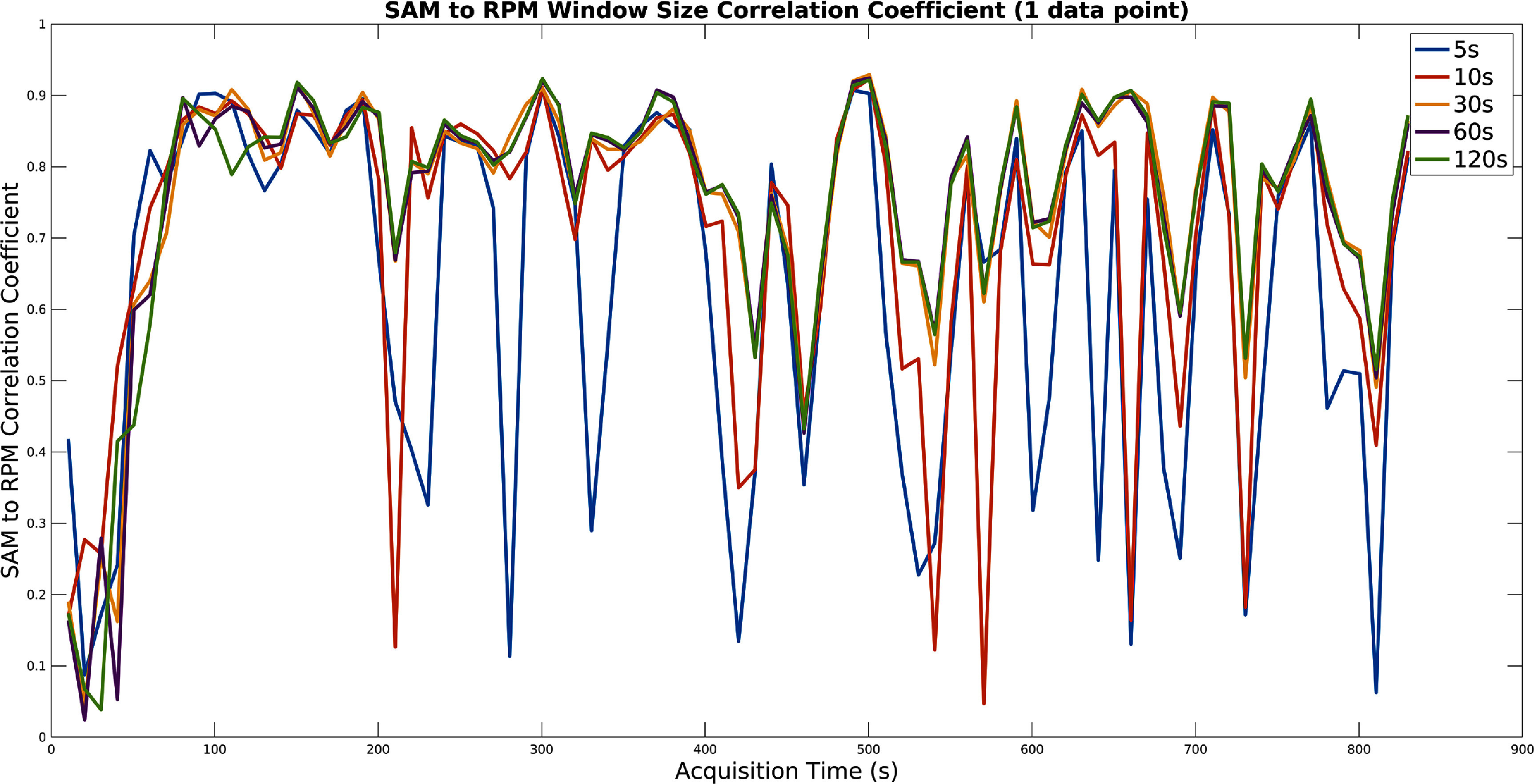
A plot showing an example of the influence of the moving windows size for the SAM method. For different fixed window sizes, the correlation of the extracted signal to the RPM is shown for the windows sliding over the whole acquisition (shown for the first acquisition of patient one). Note that 0.5 s time frames were used.

The size of each window was optimised on the training data set. The PCA method was first run with a number of fixed window sizes. The window size which gave the best signal (defined as the highest average correlation coefficient with the RPM within each window) at each time interval was selected and recorded. Example results for the moving window size can be seen in figures 2 and 3 for the PCA and SAM methods respectively.

For this method, PCA (or SAM) is applied independently on each window, and the results are averaged together (after sign correction). This is required because PCs could produce equally valid but opposite sign results. As the sign of the signal from each window is arbitrary, the overlapping allows for a common sign to be found, by comparing the correlation coefficient of the data in the overlap of neighbouring windows, and flipping windows where the correlation coefficients have opposite sign. Other methods for sign correction are possible, for example see Bertolli *et al* (2017), Feng *et al* (2018), as well as the sign choice in the algorithm 6.

### 2.4. Late time interval method

Here, a PC from a late time interval is taken, and used with data from all time intervals. The motivation behind attempting this method, was the observation that PCs from late time interval data did not vary significantly when different windows were selected. However, this was not true for early time interval data. It could be hypothesised that; because the respiratory motion should be semi-consistent throughout the acquisition, then if a PC is capturing the respiratory motion at late time intervals, it should do the same at early time intervals as well. With the current implementation, negative weights for this PC have not been considered. It may be prudent to attempt to use negative weights, due to the fact that although the respiratory motion is probably similar, the tracer distribution is very different at early time intervals.

The Late Time Interval PC method, as seen in the algorithm 3, splits the data into two channels, one which only contains later time interval data, where the radiotracer kinetics have diminished, and one which contains all the data. PCA is applied to the later time interval data only. The PC from the later time interval data can then be taken and multiplied by the channel containing all of the data, to give the weights contributing to that PC for all time points. The cutoff between early and later time interval data was determined on training data, by varying the cutoff point, and maximising the correlation coefficient between the output and RPM signal, for the first 120 s interval (between 20 s and 140 s). The cutoff determined here was 62% or approximately 520 s from the start of the acquisition. It was noted however that later cutoffs gave similar results.

**Table:** 

**Algorithm 3.** Late time interval method.
**Data:** *timeSeriesSinograms*, *lateTimeIntervalCutoff*
**Result:** *respiratorySignal*
**1** *lateTimeIntervalSeriesSinograms* = split *timeSeriesSinograms* from *lateTimeIntervalCutoff* to end
**2** *lateTimeIntervalPC* = PC from PCA for *lateTimeIntervalSeriesSinograms*
**3**
**4** **for** *sinogram* in *timeSeriesSinograms* **do**
**5** *respiratorySignal* append *sinogram* × *lateTimeIntervalPC*
**6** **end**

### 2.5. Score, select, and combine method

In this section, we describe a novel method based on a combination of previous work. The use of this method was inspired by, the observation that signals in a frequency window of respiratory motion could be seen outside of the first few PCs. Additionally, a significant number of these had far less of a frequency contribution in a frequency window of the radiotracer kinetics. However, the information contained in these PCs is ignored if only one PC is used, as in Thielemans *et al* (2011), and Bertolli (2018). This could lead to a reduced signal to noise ratio (SNR). The method therefore uses a ‘respiratory score’, and orders and combines PCs to maximise this score.

#### 2.5.1. Score and select

We developed several ways to calculate a score used for selecting PCs.

##### 2.5.1.1. Frequency based

As seen in the algorithm 4, PSD analysis (Thielemans *et al* 2011) used the PSD of the weights for each PC, to select for the PC with the highest contribution in the respiratory window. We extended this method to account for kinetic information. In our current implementation, these PSDs contain the frequency contribution of each signal, between the frequencies of 0.0 Hz and 1.0 Hz (due to sampling the input data at 2.0 Hz and the Nyquist theorem (Whittaker 1915, Nyquist 1928, Shannon 1949)). Frequency windows representing the content of information related to radiotracer kinetics, respiratory motion, and noise are defined.

**Table:** 

**Algorithm 4.** Frequency score.
**Data:** *timeSeriesSinograms*, *PC*, *kineticFrequencyWindow*, *respiratoryFrequencyWindow*,*noiseFrequencyWindow*
**Result:** *respiratoryScore*
**1** **for** *sinogram* in *timeSeriesSinograms* **do**
**2** *respiratorySignal* append *sinogram* × *PC*
**3** **end**
**4**
**5** *PSD* = absolute of FFT on *respiratorySignal*
**6**
**7** *kineticContribution* = mean value of *PSD* within *kineticFrequencyWindow*
**8** *respiratoryContribution* = mean value of *PSD* within *respiratoryFrequencyWindow*
**9** *noiseContribution* = mean value of *PSD* within *noiseFrequencyWindow*
**10**
**11** *respiratoryKineticRatio* = $\dfrac{\textit{respiratoryContribution}}{\textit{kineticContribution}}$
**12**
**13** *respiratoryNoiseRatio* = $\dfrac{\textit{respiratoryContribution}}{\textit{noiseContribution}}$
**14**
**15** *respiratoryScore* = *respiratoryKineticRatio* × *respiratoryNoiseRatio*

In an initial implementation they were defined as 0.0 Hz to 0.1 Hz, 0.1 Hz to 0.4 Hz, and above 0.4 Hz respectively (Bertolli *et al* 2017). However, it was found that the choice of respiratory window boundaries was limiting, it was both too wide (so as to encourage the mislabelling of noise), and not low enough (so as to fail on slow breathers). Thus, in the current implementation, the respiratory window is determined by first applying the Late Time Interval PC method, to acquire an initial estimate of the signal, and using this to estimate the window boundaries. A PSD of the initial estimate is acquired. The frequency which is at the mean value of the PSD is determined to be the centre of the window, and the boundaries are selected as being half the standard deviation of the PSD from this point. Half a standard deviation is used such that there is a full standard deviation between the upper and lower bounds of the window.

The contribution within each window is determined for each PC by finding the mean magnitude within the windows. Ratios are then calculated between the respiratory window and the kinetic window, and the respiratory window and the noise window, and a score determined by the product of these two values.

##### 2.5.1.2. Neural network based

**Figure 4.**
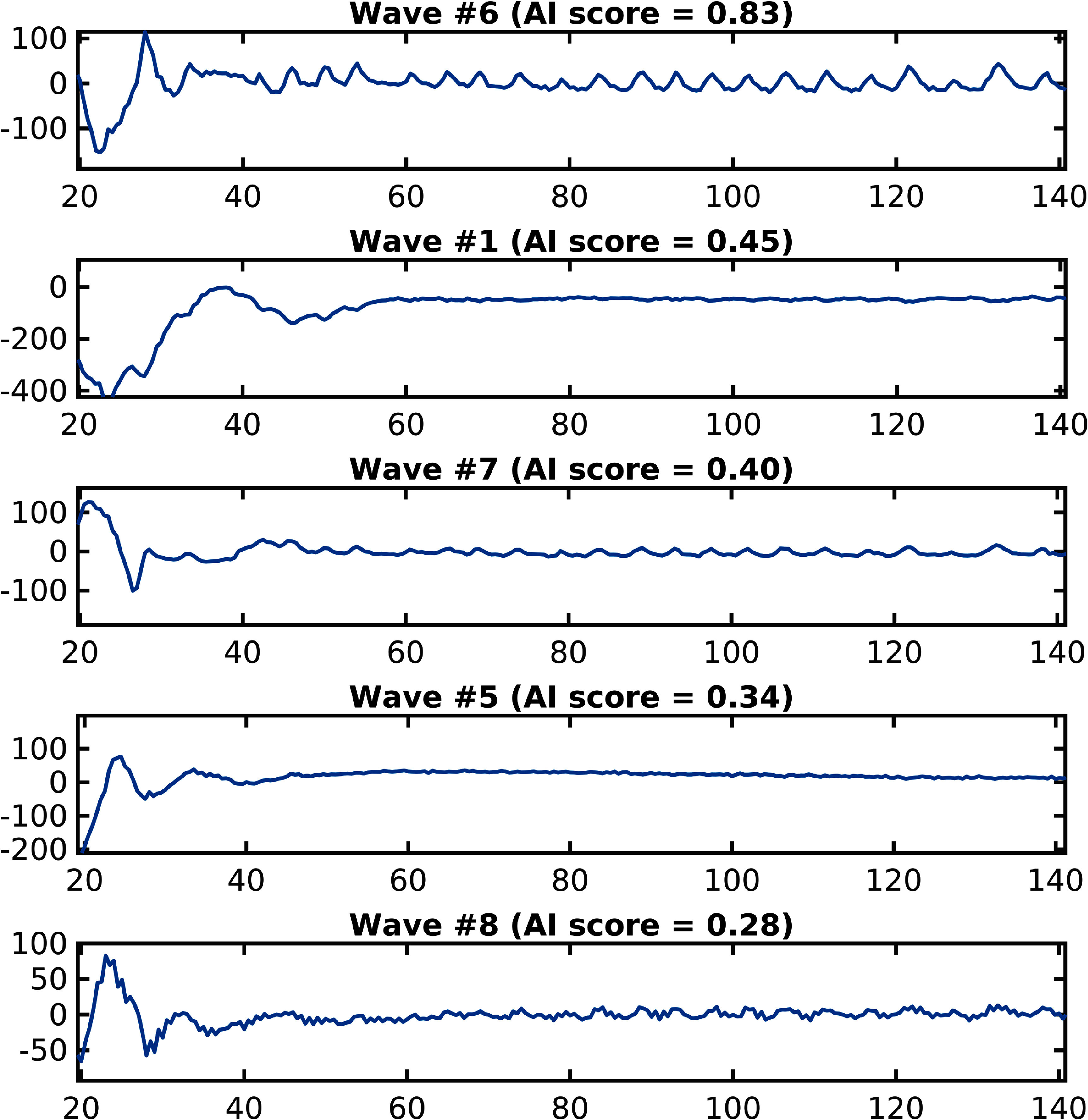
An example of the output for the neural network (NN) can be seen here testing signals extracted from the first acquisition of patient one using PCA.

A NN based scoring metric that was previously developed (Walker *et al* 2020), was tested here to remove complexity and increase robustness when compared to the frequency scoring method. The NN of Walker *et al* (2020) is a pretrained model, designed to accept a signal as input and return a score between 0.0 and 1.0. Here a higher score indicates a more respiratory like signal. To achieve this, to avoid issues with signals of different lengths, features of the signal were extracted and used as input to the NN (rather than directly inputting the signal itself). For example, one potential feature could be the PSD of the signal. The network was trained on scores predetermined by clinicians. Specifically, two clinicians scored the signals used to train this model as either being a score of 0.0, 0.5, or 1.0, the mean of the scores from the two clinicians was used as target values. Examples of the output of the NN can be seen in figure 4.

Once a score for each signal has been determined, the list of PCs is sorted following this scoring as in the algorithm 5.

**Table:** 

**Algorithm 5.** Score and select PCs.
**Data:** *timeSeriesSinograms*, *PCs*, *scoreThreshold*
**Result:** *PCs*
**1** **for** *PC* in *PCs* **do**
**2** *respiratoryScore* = get respiratory score from *PC* and *timeSeriesSinograms*
**3**
**4** **if** *respiratoryScore* $\gt$ *scoreThreshold* **then**
**5** *respiratoryScoreList* append *respiratoryScore*
**6** **else**
**7** remove *PC* from *PCs*
**8** **end**
**9** **end**
**10**
**11** sort *PCs* by *respiratoryScoreList*

#### 2.5.2. Combine

After the PCs are sorted from a high to low score, they are then iterated over, both being summed and subtracted (with a weighting, the score), and a new score is found for both resulting signals. If one of the signals increases this score, then it becomes the new best PC, and goes forward to the next iteration. PCs are both summed and subtracted to handle the arbitrary sign problem mentioned earlier in section 2.3 (Bertolli *et al* 2017).

A similar method of combining signals can be seen in Kesner and Kuntner (2010). However, the method presented here, attempts to improve on this by using a scoring metric whose behaviour better reflects the qualities of a ‘good’ signal. While in Kesner and Kuntner (2010), the standard deviation is maximised. In addition, the proposed methods compute the metric based on signals derived from PCs, as opposed to a single voxel or sinogram bin value, which should lead to noise reduction.

Please note that the algorithm 6 is described in terms of PCs. In fact, a simpler version just sums and subtracts the corresponding signals, this will give the same final signal.

**Table:** 

**Algorithm 6.** Combining PCs.
**Data:** *timeSeriesSinograms*, *PCs*
**Result:** *respiratoryPC*
**1** *respiratoryPC* = first PC in *PCs*
**2** remove *respiratoryPC* from *PCs*
**3** *respiratoryScore* = get score from *respiratoryPC* and *timeSeriesSinograms*
**4**
**5** **for** PC in *PCs* **do**
**6** *currentRespiratoryScore* = get score from PC and *timeSeriesSinograms*
**7**
**8** *scaledRespiratoryPC* = *respiratoryPC* × *respiratoryScore*
**9** *scaledCurrentPC* = PC × *currentRespiratoryScore*
**10**
**11** *sumPC* = *scaledRespiratoryPC* + *scaledCurrentPC*
**12** *subtractPC* = *scaledRespiratoryPC* - *scaledCurrentPC*
**13**
**14** *sumRespiratoryScore* = get score from *sumPC* and *timeSeriesSinograms*
**15** *subtractRespiratoryScore* = get score from *subtractPC* and *timeSeriesSinograms*
**16**
**17** **if** *sumRespiratoryScore* $\gt$ *respiratoryScore* **then**
**18** *respiratoryPC* = *sumPC*
**19** *respiratoryScore* = *sumRespiratoryScore*
**20** **else**
**21** **if** *subtractRespiratoryScore* $\gt$ *respiratoryScore* **then**
**22** *respiratoryPC* = *subtractPC*
**23** *respiratoryScore* = *subtractRespiratoryScore*
**24** **end**
**25** **end**

In our current implementation, this method is applied on all time intervals at once. It is possible to integrate this method with the Late Time Interval method in section 2.4 or the Moving Window method in section 2.3. However, this has not been demonstrated here.

## 3. Evaluation

Here, we discuss how the data was prepared for evaluation. We also present a suite of methods which can be applied generally to SSs (both from dynamic as well as potentially static acquisitions), in order to combat issues such as noise and outliers. Finally, in this section, we highlight how the methods in section 2 will be evaluated in section 4.

### 3.1. Data preparation

Time of flight (TOF) data were unlisted into low spatial resolution sinograms, each with a time frame duration of 500 ms, using the GE PetToolbox, following Bertolli *et al* (2017), resulting in sinograms with dimensions $95\times16\times47\times11$ (radial positions × angles × transaxial plane × TOF). To extract respiratory variation, the sampling rate of the PET sinograms was chosen as 2 Hz, so as to attempt to mitigate the effect of cardiac motion (Bertolli 2018).

Data was pre-processed element-wise by first applying a Freeman-Tukey transformation (Freeman and Tukey 1950), followed by a Yeo–Johnson power transformation (Yeo and Johnson 2000), to transform the Poisson distributed data to be more Gaussian-like.

In the current implementation, these transformations are applied element-wise on the (Poisson distributed) TOF sinogram *S_p_*

\begin{equation*} S_g = \mathrm{YJ_\lambda}\left(\mathrm{FT}\left(S_p\right)\right).\end{equation*}

The Freeman–Tukey transformation is defined as

\begin{equation*} \mathrm{FT}\left(X\right) = \sqrt{X + 1} + \sqrt{X}.\end{equation*}

The Yeo–Johnson power transformation is defined as

\begin{equation*} \mathrm{YJ_\lambda}(X) = \begin{cases} ((X + 1)^\lambda - 1) / \lambda &amp; \quad \textrm{if } \lambda \neq 0 \textrm{, } X \unicode{x2A7E} 0 \\ \log(X + 1) &amp; \quad \textrm{if } \lambda = 0 \textrm{, } X \unicode{x2A7E} 0 \\ -\left[(-X + 1)^{(2 - \lambda)} - 1)\right] / (2 - \lambda) &amp; \quad \textrm{if } \lambda \neq 2 \textrm{, } X < 0 \\ -\log(-X + 1) &amp; \quad \textrm{if } \lambda = 2 \textrm{, } X < 0. \end{cases}\end{equation*}

The *λ* parameter is determined by minimising the Kullback Leibler Deviation (KLD) between normal distributions and the transformed distribution (Yeo and Johnson 2000). In this paper a single *λ* was determined from all of the data, although it would be feasible to find different *λ* values for every element in the sinogram.

The impact of including the Yeo-Johnson power transformation can be seen in table 1.

**Table 1.** This table shows the correlation coefficient between the result of the Score, Select, and Combine using NN scoring and the RPM for the train dataset where a number of pre- and post-processing methods have been applied to the data. Each new line represents the addition of this method, therefore the last element includes all previous pre- and post-processing. This is for the Freeman-Tukey transform, the Yeo–Johnson transform, the incorporation of a mask to remove low count areas of the sinogram, smoothing and downsampling the sinogram and smoothing the signal, and parallel compression.

Correlation coefficient with RPM	20 s–840 s	20 s–140 s
Freeman–Tukey	0.783	0.703
Yeo–Johnson	0.790	0.747
Mask	0.826	0.824
Smoothing and downsampling	0.826	0.825
Parallel compression	0.830	0.839

It was previously found through experimentation, that Gaussian smoothing of the resulting sinograms can improve results, especially in the case of the SAM methods (Thielemans *et al* 2013). In the current implementation, further downsampling was performed post-smoothing to reduce memory usage, and increase computational speed. Linear interpolation was used as it was shown in a preliminary investigation to give satisfactory results at little computational cost. Although block reduction (by summing patches of data of size four or eight etc) is more computationally efficient, and may potentially have benefits with regards to noise over linear interpolation. However, block reduction is limited in what downsampling factors can be used, while interpolation is not. We use linear interpolation here to reduce the matrix size to a size necessary to represent the highest frequency information present in the data post-smoothing. The effect this inclusion makes on the end result can be seen in table 1.

Finally, it has been found that the introduction of a mask to further aid in the reduction of noise is beneficial (Thielemans *et al* 2011). The mask itself is defined as being true for any value, in the sinogram, above a predetermined threshold. Values not in the mask are removed prior to further execution, this is because these values can be assumed to mostly be noise. Note that a mask can also be used to eliminate parts of the data potentially affected by non-respiratory movement (Bertolli 2018), but this has not been implemented in the current work. Again, the impact of this inclusion can be seen in table 1.

Values for the Gaussian smoothing, and the threshold of the mask, were determined using a grid search on a randomly selected subset of the data (specifically three patients). This data was then not used as part of any final evaluation, as was stated in section 2.1. The Gaussian smoothing sigma for the PCA based methods were 1.0, 0.5, and 2.0, for SAM based methods they were 1.0, 3.0, and 1.0 (this is for the sinogram radial positions, angles, and transaxial planes respectively). The mask threshold is selected programmatically, such that it removes the bottom 5% of values. The same mask is used for every time point. From an examination of the masks used for the test dataset, because a threshold value as low as 5% is used, it appears that the mask is only removing parts of the background.

### 3.2. Post-processing

Regardless of the method used, there are still some effects of the radiotracer kinetics to be expected at early time intervals, and noise throughout. Thus, a method is proposed here to aid with the remaining radiotracer kinetics, and smoothing to help with noise in the extracted SS. The same post-processing is used regardless of the method to extract the SS.

#### 3.2.1. Parallel compression

Firstly there is, what shall be referred to as ‘parallel compression’. This is a method borrowed from audio engineering (appearing notably in Dolby A noise reduction). The signal is split into two channels, one has its dynamic range reduced (through a process such as compression), while the other passes unchanged, before they are averaged back together (Izhaki 2012). This has the effect of reducing large differences in the dynamic range of the signal (for instance caused by tracer kinetics or some kind of drift), without losing a lot of breath to breath variability, compared to directly using the phase of the signal (if applied to respiratory SSs) (Lamare *et al* 2022). For a more detailed explanation of the implementation please see the appendix.

#### 3.2.2. Outlier removal

Even though most of the large changes in intensity are remedied by parallel compression, some momentary spikes are still apparent. Thus, outliers are removed where they are outside a threshold of the quartile of the signal, and new values are interpolated.

#### 3.2.3. Smoothing

Finally, smoothing is applied through the use of a bandpass filter (specifically a sinc filter), followed by a Savitzky-Golay filter (Savitzky and Golay 1964). A bandpass filter is used to remove frequencies in the signal outside of the respiratory window, and the Savitzky–Golay filter is used to promote local smoothness.

The bandpass filter is defined as

\begin{equation*} h_{\textrm{BPF}}\left(t\right) = 2B_H\textrm{sinc}\left(2B_Ht\right) - 2B_L\textrm{sinc}\left(2B_Lt\right)\end{equation*}

where in equation (5) *h*_*BPF*_ is the bandpass function, *t* is the time distributed variable, *B_H_* is the upper bound and *B_L_* is the lower bound of the bandpass filter. The bandpass filter is implemented using a truncated sinc kernel. A polynomial order of three and a window length of five was used with the Savitzky-Golay filter, determined through a grid search on the training dataset.

The impact of the inclusion of the above methods on the correlation of the Score, Select, and Combine method with NN based scoring with the RPM can be seen in table 1.

### 3.3. Evaluation methods

For evaluation of the results, the correlation coefficient of each SS between each method and the RPM, for all acquisitions in the test dataset, has been calculated. The correlation coefficient has been calculated for both the first 120 s (ignoring the first 20 s), and also the entire acquisition (between 20 s and 840 s). A statistical analysis using a mixed effects model has also been included.

All methods were compared to conventional PCA. We also included results for the Moving Window SAM method as this approximates KRG. While the conventional and Late Time Interval methods can also be implemented using SAM, corresponding results are not shown here.

As stated earlier in section 2.1, parameters for the methods have been selected using a grid search on a randomly selected subset of the data (specifically three patients). This data was then not used as part of any final evaluation. The parameters were optimised by maximising the correlation coefficient between the SS and the RPM for the first 120 s of usable data (between 20 s and 140 s), due to there being initially no counts in the FOV.

## 4. Results

**Figure 5.**
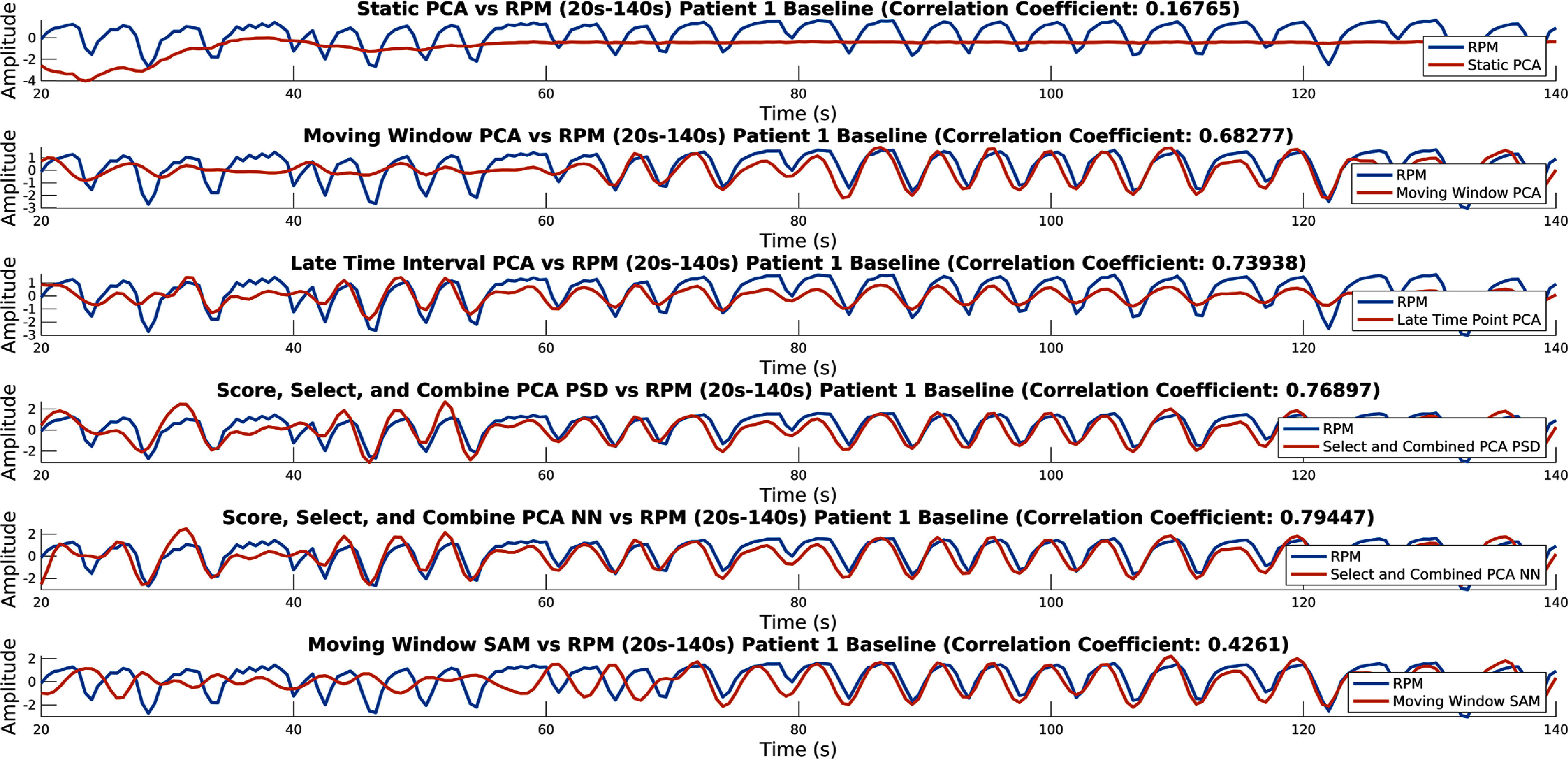
Output of each method compared to the RPM for the first usable 120 s (between 20 s and 140 s) (taken for the first acquisition of patient one). This is for conventional PCA, Moving Window PCA, Late Time Interval PC, Score, Select, and Combine using frequency and NN scoring, and the Moving Window SAM method.

A plot showing for each method its output, compared to the RPM, for the first 120 s (between 20 s and 140 s) (taken for the first acquisition of patient one), can be seen in figure 5. From a visual analysis, it can be observed that the conventional PCA method has failed, post normalisation, it appears almost as if there is no variation in the signal at early time intervals. Both Moving Window methods show, towards the end of the plot, that they can extract a signal. However, it takes until between 60 s and 80 s for both methods to begin to pick up the signal. For the SAM based method, it appears as if the sign determination method has failed before 80 s, regardless though the method still cannot extract a signal before 60 s. The Late Time Interval, Score, Select, and Combine using frequency and NN scoring methods, all appear to be able to extract a usable signal right down to 20 s (around when counts begin to appear in the FOV). The magnitude of the signal post 80 s more closely matches the RPM (or in comparison to before 80 s) for both Score, Select, and Combine methods than for the Late Time Interval method.

**Figure 6.**
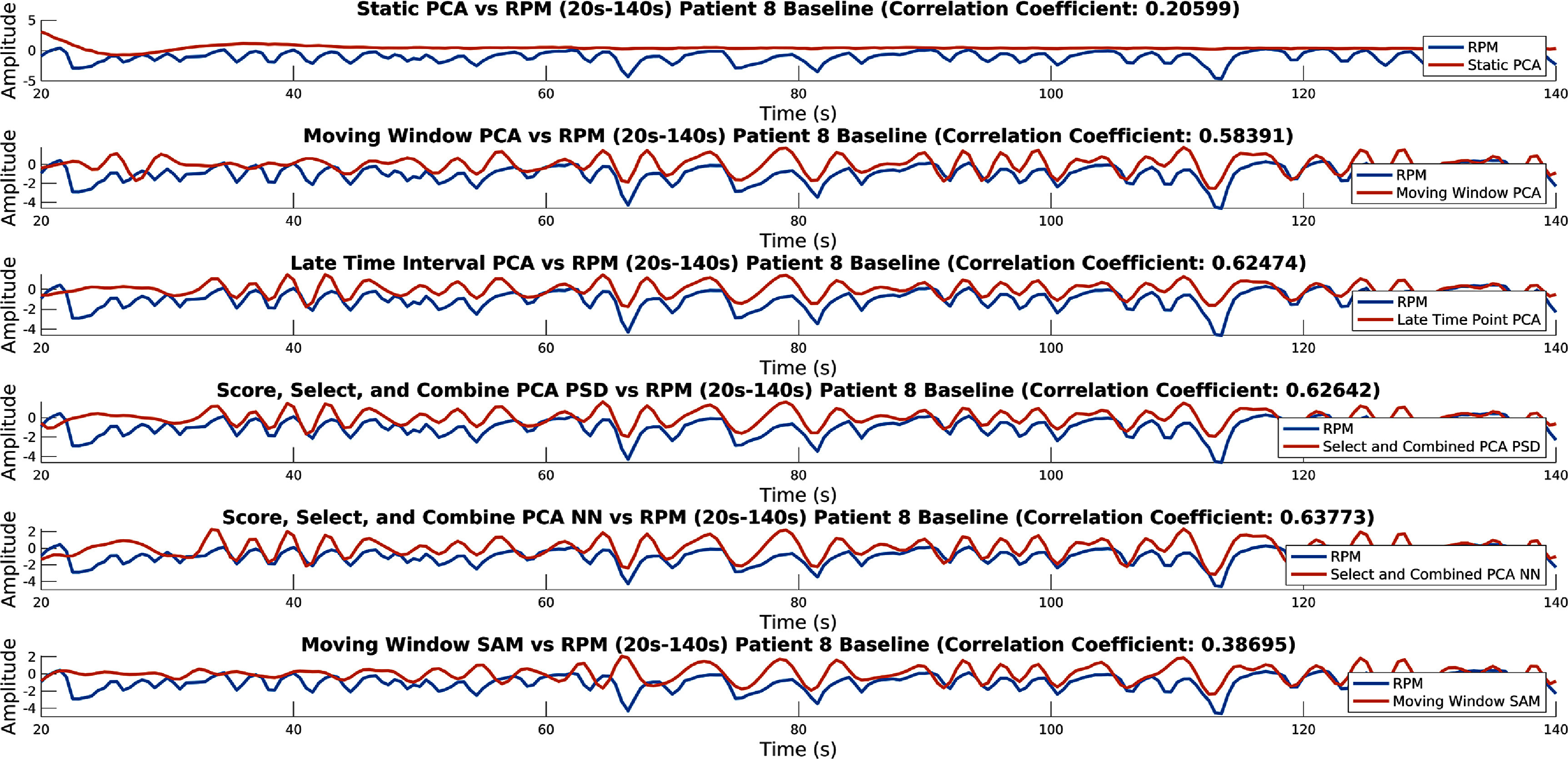
Output of each method compared to the RPM for the first usable 120 s (between 20 s and 140 s) (taken for the first acquisition of patient eight). This is for conventional PCA, Moving Window PCA, Late Time Interval PC, Score, Select, and Combine using frequency and NN scoring, and the Moving Window SAM method.

A plot showing for each method its output, compared to the RPM, for the first 120 s (between 20 s and 140 s) (taken for the first acquisition of patient eight) can be seen in figure 6. Similar results as for in figure 5 are repeated in figure 6 (although all methods match the RPM worse than in figure 5). This acquisition was selected to be shown due to it being a difficult trace to extract. Regardless, the Late Time Interval PC and Score, Select, and Combine methods appear to have extracted a signal early into the acquisition (from about 35 s on this patient and acquisition).

**Figure 7.**
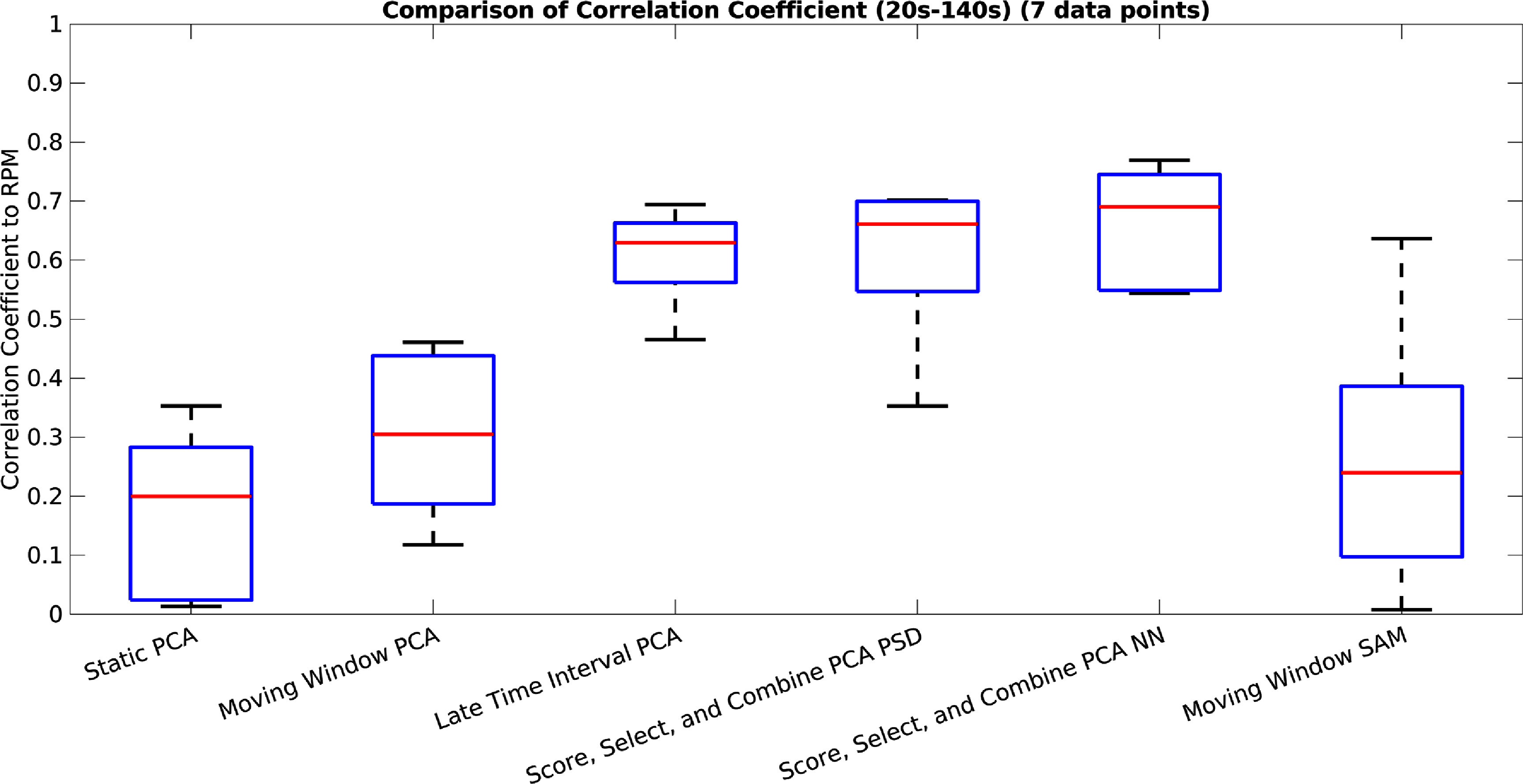
A box plot showing for each method its correlation coefficient to the RPM for the first usable 120 s (between 20 s and 140 s) (taken for the seven test acquisitions). This is for conventional PCA, Moving Window PCA, Late Time Interval PC, Score, Select, and Combine using frequency and NN scoring, and the Moving Window SAM method.

**Figure 8.**
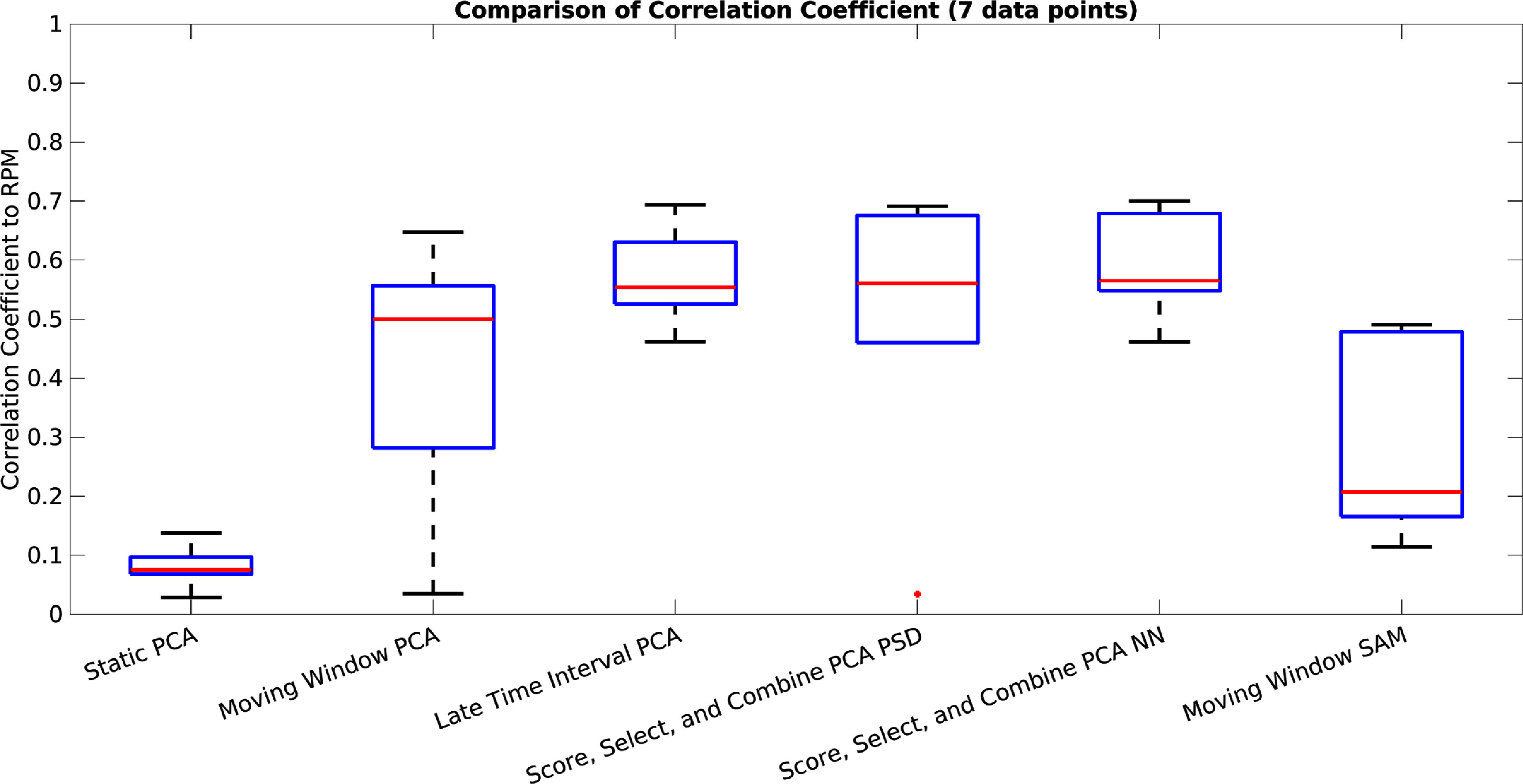
A box plot showing for each method its correlation coefficient to the RPM for the entire acquisition (taken for the seven test acquisitions). This is for conventional PCA, Moving Window PCA, Late Time Interval PC, Score, Select, and Combine using frequency and NN scoring, and the Moving Window SAM method.

A box plot showing for each method its correlation coefficient to the RPM for both the first 120 s (between 20 s and 140 s), and also for the entire acquisition (taken for seven acquisitions), can be seen in figures 7 and 8. The correlation coefficient for the conventional PCA method is low and the method is not usable. The correlation coefficients for both the Moving Window methods are roughly around 0.5, indicating that the Moving Window method is beneficial regardless of the method used to extract the signal for each window. However, again here, the correlation coefficient is lower than is acceptable. The results from the Late Time Interval, Score, Select, and Combine using frequency and NN scoring methods, all show correlation coefficients around 0.6 or higher, for both the early time interval as well as for all data. The Score, Select, and Combine methods show marginally higher correlation coefficient than the Late Time Interval method, and the NN shows slightly higher correlation coefficient than the frequency based scoring.

**Figure 9.**
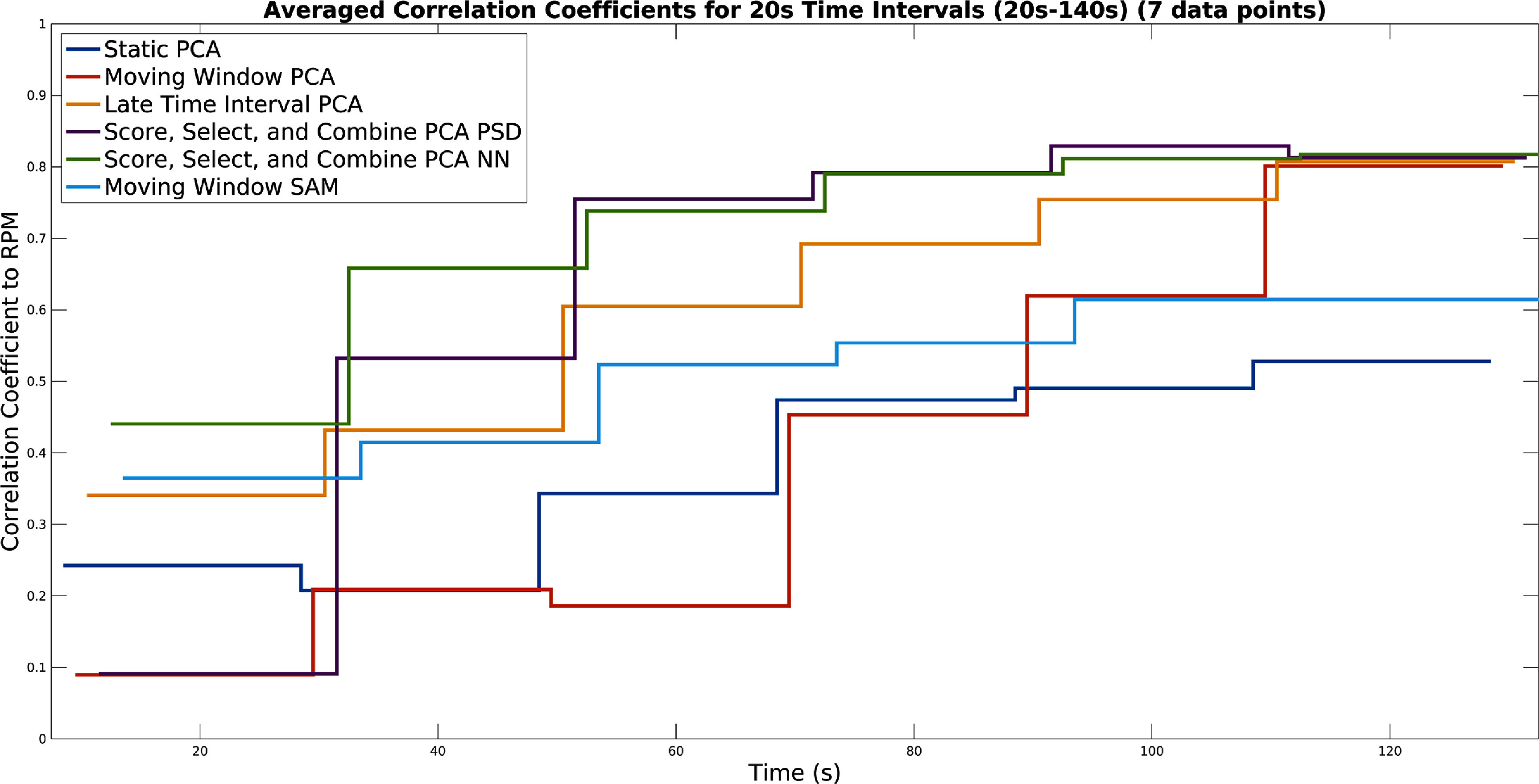
Correlation coefficients to the RPM for the first 120 s in 20 s intervals (between 20 s and 140 s) (taken as a mean for all data sets). This is for conventional PCA, Moving Window PCA, Late Time Interval PC, Score, Select, and Combine using frequency and NN scoring, and the Moving Window SAM method. The stair plots are staggered for the different methods for visual clarity.

A plot showing for each method the evolution of the correlation coefficient with the RPM over time, for the first 120 s, by computing it in 20 s intervals, can be seen in figure 9. It can be observed, that on average across all data sets all methods struggle to produce usable results at the very beginning of the acquisition (around when counts begin to appear in the FOV). However, it is also apparent that on average both Score, Select, and Combine methods robustly begin to produce results, which closely match the RPM, as evidenced by a reasonable correlation past the first 40 s, on most acquisitions. The Moving Window method appears to perform well in the first interval.

**Figure 10.**
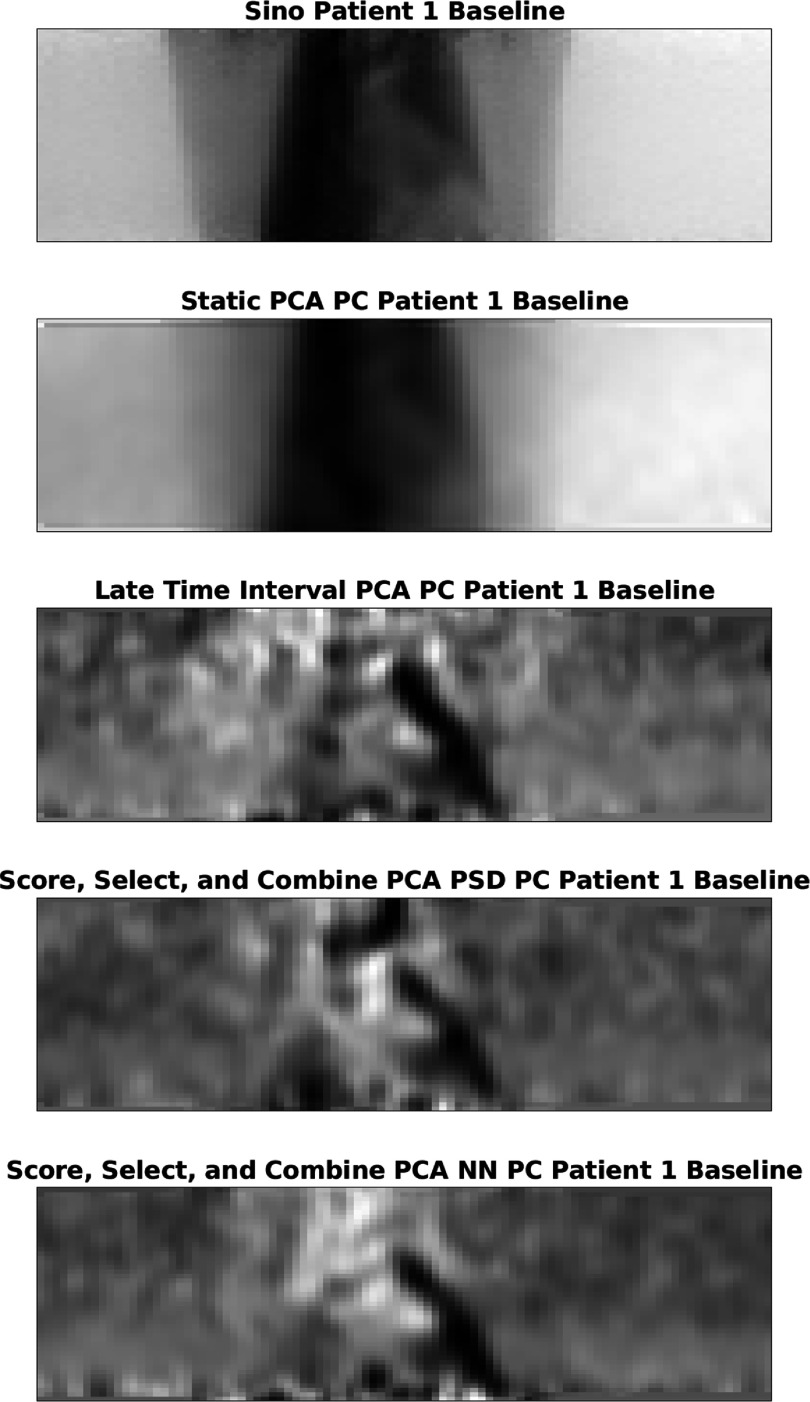
A plot showing a single ‘view’ of the original PET data (top) as well as the PCs used to generate the output signal for the conventional PCA, Late Time Interval PCA and the two Score, Select, and Combine methods (taken for the first acquisition of patient one).

A plot showing the component (or their combination) used to generate the output signal, can be seen in figure 10. One advantage of the sinogram-based methods is that the PC (or signed mask for SAM) can be visualised to see how it corresponds to anatomy and tracer uptake. In figure 10 it can be seen that the conventional PCA method returns a PC which closely resembles the input data, leading to the conclusion that the variation in the selected PC is dominated by the kinetics. The other methods produce a PC which is more related to edges of internal structures, where respiratory movement occurs. A visual inspection indicates that the least confounding variation, and noise, is included in the Score, Select, and Combine using the NN scoring method. Curiously, it appears that the Late Time Interval, and the Score, Select, and Combine using the NN scoring method return very similar distributions. However, the Score, Select, and Combine using the frequency scoring method also returns a high value region in tissue at the top of the image.

We utilised a mixed-effects model to analyse the differences between various methods and RPM measurements across all subjects. This model incorporated ‘method’ as a fixed effect and treated subjects as a random effect, to account for inter-subject variability. We found that the comparisons between each method and RPM were not statistically significant, with all p-values $ > 0.05$, see table 2. While this may suggest that, within the constraints of our analysis and available data, there was no evidence to support significant differences between any methods in comparison to RPM measurements, given the small number of subjects, it is important to consider that this analysis may be considerably underpowered.

**Table 2.** This table shows the *p*-values acquired as part of a statistical analysis of the test dataset using a mixed-effects model. Before applying the mixed-effects model the signals were first transformed to frequency space using FFT. One values between 0.05 Hz and 0.45 Hz were used. This is for conventional PCA, Moving Window PCA, Late Time Interval, Score, Select, and Combine using frequency and NN scoring, and the Moving Window SAM method.

Mixed-effects model	*p*-value
Static PCA	0.183
Moving Window PCA	0.483
Late Time Interval PCA	0.548
Score, Select, and Combine PCA PSD	0.649
Score, Select, and Combine PCA NN	0.908
Moving Window SAM	0.373

It is noteworthy to mention that we observed that the Late Time Interval and Score, Select, and Combine using frequency and NN scoring methods exhibited the highest p-values. This may suggest a lesser degree of difference from RPM. However, such interpretations should be approached with caution and not be taken as conclusive evidence of similarity.

Finally, results of applying the Score, Select, and Combine using NN scoring method to data where the RPM could not by synced with the list mode data can be seen in 1.

## 5. Discussion

This paper introduces several methods for DD extraction of a respiratory signal from dynamic PET data. To the best of our knowledge, this appears to have only been attempted in Schleyer *et al* (2014). Data used here are from a [^18^F]-FDG study on patients with IPF, while in the latter paper, Nitrogen-13 Ammonia ([^13^N]-NH3) data was used to evaluate the proposed KRG method.

The work presented suffers from several limitations. Firstly, the data used all originates from the same study, using the same procedure, the same radiotracer, and acquired on the same scanner. In order to better validate the generalisability of the method it would be positive to test on data acquired on different scanners and using different radiotracers. Additionally, from the data acquired, only a subset of this data is usable due to issues during acquisition. The number of participants is limited, it would be beneficial to test the methods on a larger sample of patients. Furthermore, it would be beneficial for the data to include both a larger number of non-complex and complex breathers to better test the limitations of the methods.

**Figure 11.**
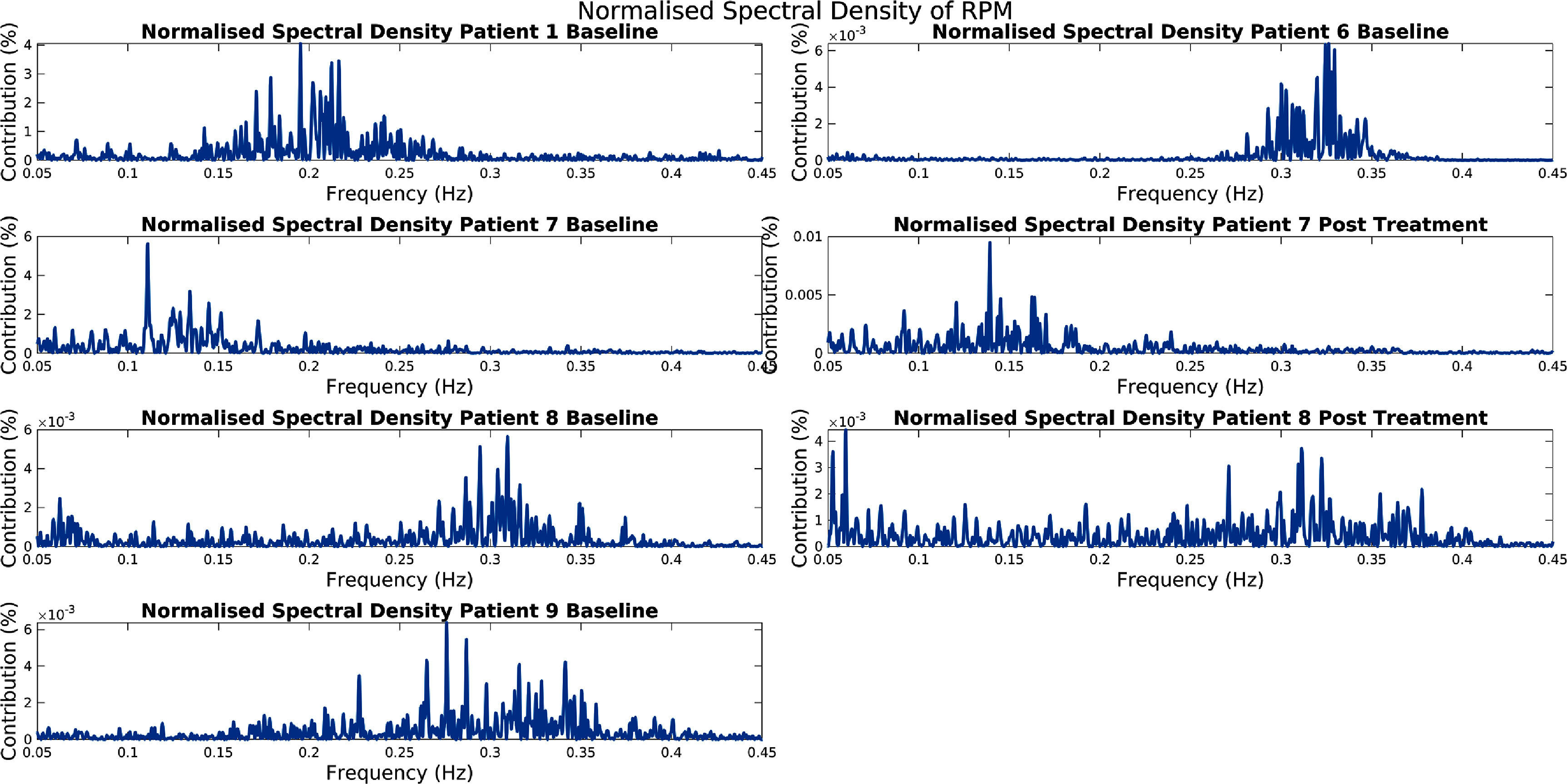
The result of applying FFT to the RPM signals for the first usable 120 s (between 0.05 Hz and 0.45 Hz) (for the seven test acquisitions). Notice that four of the seven have peaks very close to or outside the lower boundary of the resiratory window. Also notice that two of the seven have very wide frequency responses, which would be difficult for the automatic selection of a respiratory frequency window.

**Figure 12.**
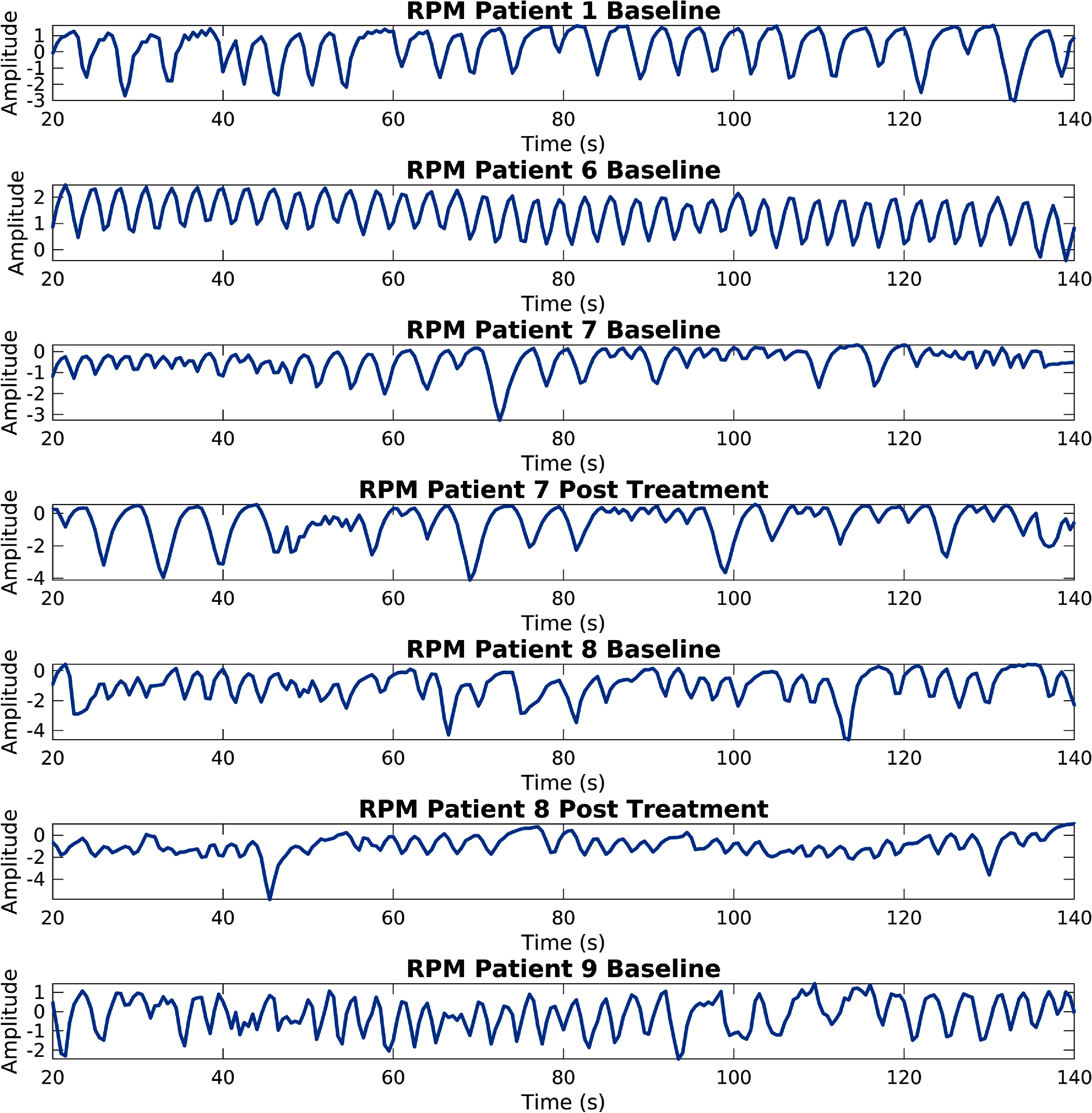
RPM signals for the first usable 120 s (between 20 s and 140 s) (for the seven test acquisitions). Notice that only the first acquisition of patient one and patient six shows a steady trace with an average frequency, every other trace shows variable breathing or artefacts.

An additional concern is the point at which the methods may fail, for patients who exhibit abnormal breathing patterns. For instance, extremely slow breathers will breathe at a rate less than 0.1 Hz, which in the case of the Score, Select, and Combine method using the frequency scoring method and a fixed frequency respiratory window would be considered to be radiotracer kinetics. Furthermore, when using a non-fixed respiratory window this method struggles with patients who breathe less regularly, as the window is expanded to include parts of the kinetics and noise (this is shown in figure 11). The discrepancy between the results for the SAM Moving Window method, presented here, and the KRG ones, shown in Schleyer *et al* (2014), could also be attributed to this complexity. Many of the patients breathed at varying rates, stopped breathing during acquisition, or breathed unusually fast or slow (this is shown in figure 12). This was probably due to the data being acquired for an IPF study.

The presented method includes several pre- and post-processing steps. Although these have been shown to be beneficial on the training dataset, table 1, the impact of some steps is relatively small. It remains to be determined on a larger dataset if some of these steps could be removed.

A limitation of the concept of using SSs, at all, in the pursuit of the motion correction of respiratory motion, is the transform from a one dimensional (1D) SS to the three dimensional (3D) motion. There are methods for this transformation, such as one using a motion model (MM) parameterised by the SS (McClelland *et al* 2013, 2017). However, they are not trivial, and in the case where a 1D SS is used, limit breath to breath variability and hysteresis to being periodic (Whitehead *et al* 2021). There are beginning to be methods for motion correction which are conditioned on features of the acquisition rather than a SS (Huang *et al* 2023, 2024).

## 6. Conclusion

We have presented and evaluated several methods for extraction of a respiratory signal from dynamic PET data. Results from a visual comparison of early time interval output signals compared to the RPM, quality of PC, and correlation coefficient of the output signals to the RPM, indicates that the Late Time Interval and both Score, Select, and Combine methods are more robust and afford higher quality signals than Moving Window methods. The results also indicate that both Score, Select, and Combine methods can give a higher correlation coefficient earlier than the Late Time Interval method. Scoring using the NN shows slightly higher correlation coefficients than the frequency based scoring.

In the future, research will focus on further development of the method, including optimisation of the NN scoring method. In the next stage, these methods will be applied to the task of implementing advanced respiratory motion correction on dynamic PET data.

## Figures and Tables

**Figure 13.**
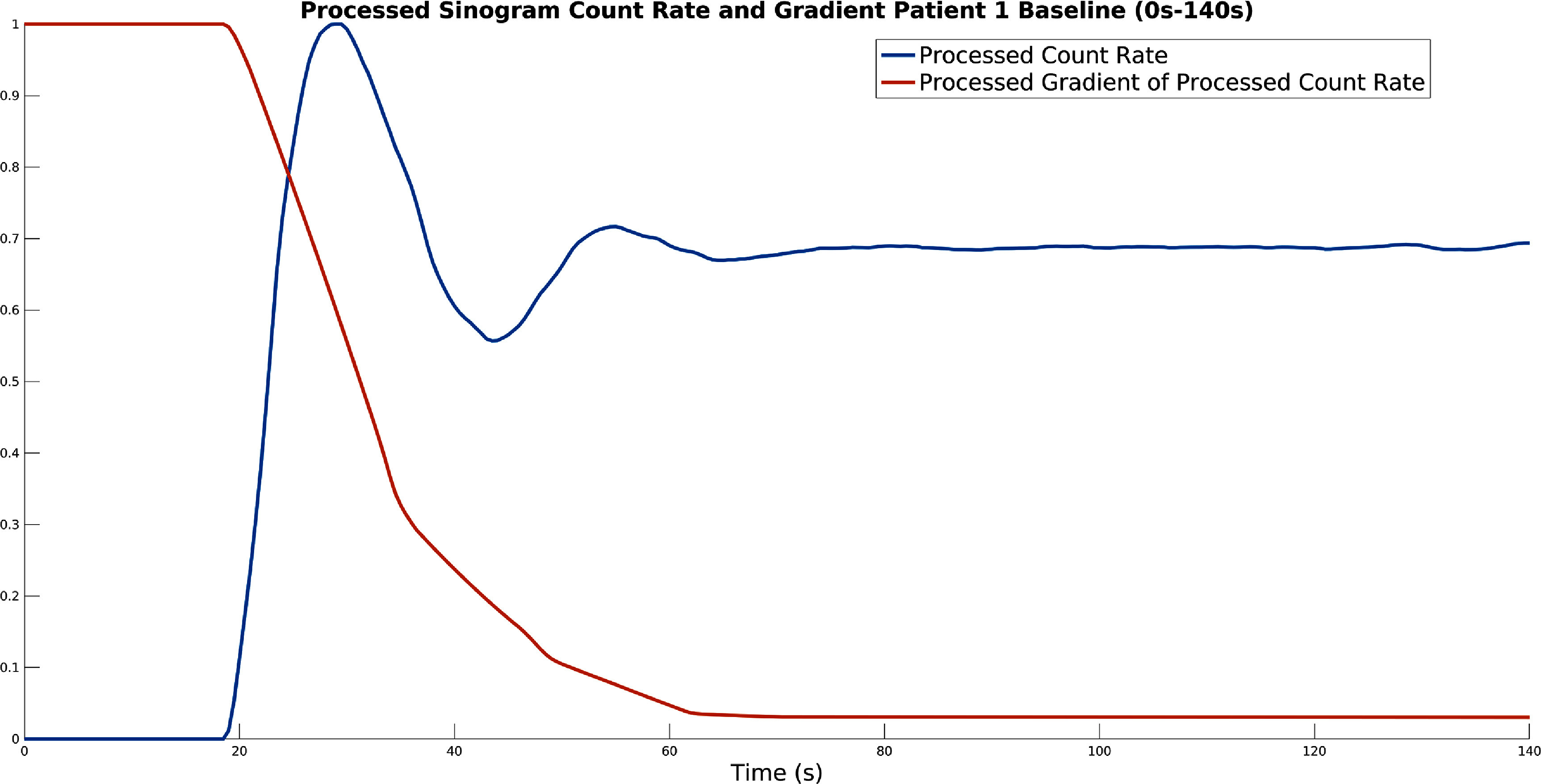
This figure shows an example of the processed count rate and processed gradient of the processed count rate, between 0 s and 140 s, which are used as part of the parallel compression post-processing step. These have been taken for the first acquisition of patient one. Here the processed count rate is normalised between zero and one by subtracting by the minimum and dividing by the maximum value of the signal.

## Data Availability

The data cannot be made publicly available upon publication because they contain sensitive personal information. The data that support the findings of this study are available upon reasonable request from the authors.

## Appendix

### A.1. Parallel compression

This appendix contains a detailed description of what is presented briefly in section 3.2.1. This method attempts to limit the dynamic range of a signal proportionally, in time, to how much that signal is expected to be affected by artefacts. The signal is duplicated into two channels, one of these channels is then split temporally into a series of small moving windows. These windows are 20 s in size and overlap by their length minus one (the stride size is one). The values in each window are then standardised independently, the windows are averaged back together, before being combined with the unadulterated channel following the weighting seen in figure 13. An example of the algorithm can be seen in the algorithm 7.

**Table:**

**Algorithm 7.** Parallel compression.
**Data:** *timeSeriesSinograms*, *respiratorySignal*, *windowSize*
**Result:** *respiratorySignal*
**1** **for** *index* in length of *timeSeriesSinograms* - *windowSize* **do**
**2**
**3** *currentRespiratorySignal* = *respiratorySignal* for data between *index* and *index* + *windowSize*
**4**
**5** *currentRespiratorySignal* = *currentRespiratorySignal* − mean of *currentRespiratorySignal*
**6** *currentRespiratorySignal* = $\dfrac{\textit{currentRespiratorySignal}}{\mathrm{standard \ deviation\ of\ }\textit{currentRespiratorySignal}}$
**7**
**8** *windowSignal* = fill with NaNs to *index*
**9** *windowSignal* append *currentRespiratorySignal*
**10** *windowSignal* append NaNs to length of *timeSeriesSinograms*
**11**
**12** *respiratorySignals* append *windowSignal*
**13**
**14** **end**
**15**
**16** *lowDynamicRangeSignal* = mean of *signals* ignoring NaNs
**17**
**18** *weighting* = get weighting from *timeSeriesSinograms*
**19**
**20** *lowDynamicRangeSignal* = *lowDynamicRangeSignal* × *weighting*
**21** *respiratorySignal* = *respiratorySignal* × (1 – *weighting*)
**22**
**23** *respiratorySignal* = *respiratorySignal* + *lowDynamicRangeSignal*

The channels are combined following the gradient of the count rate. An example of the signals involved can be seen in figure 13, and an example of the algorithm can be seen in the algorithm 8.

• Firstly, the count rate over time is found by summing all elements of each sinogram. A moving average smoothing is then applied to this processed count rate signal with a size of 5 s.
• Secondly, the gradient of the processed count rate is taken. The absolute of this signal is used, this is because we are interested in the change in gradient (but not the direction). Next the processed gradient of the processed count rate signal is iterated over from its last value to its first. If the value decreases, it is replaced by interpolation, using the previous value and the next value that is larger than the previous value. Finally a (centred) moving average smoothing is applied to the signal with a size of 20 s, and the signal is normalised between zero and one by subtracting by the minimum and dividing by the maximum value of the signal.
• Thirdly, the low and high dynamic range respiratory signals are summed, using the magnitude of the processed gradient of the processed count rate signal as a weighting. At time intervals where the magnitude of the processed count rate signal is greater, more of the compressed/standardised signal is summed, compared to the non-compressed/non-standardised signal. The total weighting is always equal to one, to maintain the scale. To achieve this, the low dynamic range respiratory signal is multiplied directly by the processed gradient of the processed count rate signal, and the high dynamic range respiratory signal is multiplied by one minus the processed count rate signal (before both respiratory signals are summed together).

**Table:**

**Algorithm 8.** Extract parallel compression weighting.
**Data:** *timeSeriesSinograms*
**Result:** *processedGradientOfProcessedCountRate*
**1** **for** *sinogram* in *timeSeriesSinograms* **do**
**2** *countRate* append sum of *sinogram*
**3** **end**
**4**
**5** *processedCountRate* = moving average of *countRate*
**6**
**7** *gradientOfProcessedCountRate* = gradient of *processedCountRate*
**8**
**9** *processedGradientOfProcessedCountRate* = absolute of *processedGradientOfProcessedCountRate*
**10**
**11** *processedGradientOfProcessedCountRate* = reverse of *processedGradientOfProcessedCountRate*
**12** *currentMaximumValue* = 0.0
**13**
**14** **for** *value* in *processedGradientOfProcessedCountRate* **do**
**15** **if** *value* > *currentMaximumValue* **then**
**16** *currentMaximumValue* = *value*
**17** **else**
**18** *value* = NaN
**19** **end**
**20** **end**
**21**
**22** *processedGradientOfProcessedCountRate* = reverse of *processedGradientOfProcessedCountRate*
**23** linear interpolate NaNs in *processedGradientOfProcessedCountRate*
**24**
**25** *processedGradientOfProcessedCountRate* = centred moving average of *processedGradientOfProcessedCountRate*
**26**
**27** *processedGradientOfProcessedCountRate* = *processedGradientOfProcessedCountRate* − minimum of*processedGradientOfProcessedCountRate*
**28** *processedGradientOfProcessedCountRate* = $\dfrac{\textit{processedGradientOfProcessedCountRate}}{maximum\ of\ \textit{processedGradientOfProcessedCountRate}}$
